# Decoding glycomics with a suite of methods for differential expression analysis

**DOI:** 10.1016/j.crmeth.2023.100652

**Published:** 2023-11-21

**Authors:** Jon Lundstrøm, James Urban, Daniel Bojar

**Affiliations:** 1 Department of Chemistry and Molecular Biology, University of Gothenburg, 41390 Gothenburg, Sweden; 2 Wallenberg Centre for Molecular and Translational Medicine, University of Gothenburg, 41390 Gothenburg, Sweden

**Keywords:** glycomics, bioinformatics, computational biology, carbohydrate, glycan, statistics

## Abstract

Glycomics, the comprehensive profiling of all glycan structures in samples, is rapidly expanding to enable insights into physiology and disease mechanisms. However, glycan structure complexity and glycomics data interpretation present challenges, especially for differential expression analysis. Here, we present a framework for differential glycomics expression analysis. Our methodology encompasses specialized and domain-informed methods for data normalization and imputation, glycan motif extraction and quantification, differential expression analysis, motif enrichment analysis, time series analysis, and meta-analytic capabilities, synthesizing results across multiple studies. All methods are integrated into our open-source glycowork package, facilitating performant workflows and user-friendly access. We demonstrate these methods using dedicated simulations and glycomics datasets of *N*-, *O*-, lipid-linked, and free glycans. Differential expression tests here focus on human datasets and cancer vs. healthy tissue comparisons. Our rigorous approach allows for robust, reliable, and comprehensive differential expression analyses in glycomics, contributing to advancing glycomics research and its translation to clinical and diagnostic applications.

## Highlights

• A generalizable framework for differential glycomics expression analysis
• Sensitive detection of differences paired with statistical rigor
• Enables flexible motif-level analysis
• Integration within glycowork facilitates performant and customized workflows

## Motivation

Glycomics data analysis is challenging due to glycan complexity and data quality. Methods to address issues such as missing data, variance heterogeneity, multiple testing correction, and motif-level analysis are needed to empower sensitive, rigorous insights from glycomics datasets. This work develops computational methods and workflows to enable robust differential expression analysis in glycomics research. By integrating a suite of analytical capabilities into the glycowork package, we aim to advance glycomics research and translation by making state-of-the-art analysis and data interpretation accessible to the field.

## Graphical abstract

**Figure:**
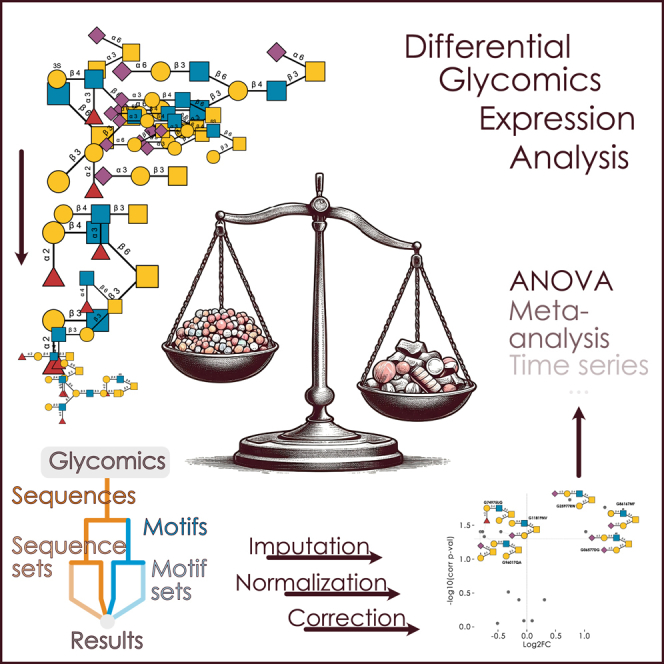

## Teaser

Lundstrøm et al. present computational methods to enable robust differential expression analysis in glycomics research, on the sequence and substructure levels. Integrated into the glycowork package, their framework empowers sensitive, rigorous insights from glycomics datasets by addressing issues such as missing data and multiple testing corrections.

## Introduction

Glycomics, predominantly assessed via mass spectrometry,^1^ plays crucial roles in understanding the biological processes in which glycans or complex carbohydrates are involved, such as disease pathogenesis.^2^^,^^3^ Glycans, linked to proteins or lipids, are involved in biological events, such as cell signaling, the immune response, and pathogen-host interactions,^2^ and can interact with proteins via specific substructures,^4^ such as the Lewis antigens.^5^ Besides functional importance, characteristic structural differences also exhibit diagnostic potential, with elevated expression of Sialyl-Lewis structures in various forms of cancer,^6^ among others.^7^ Therefore, the precise elucidation of glycan structures and their differential expression across different conditions is paramount for both fundamental and clinical research, as (for instance) the assignment of a differentially expressed Sialyl-Lewis × Epitope requires both structural annotation to the linkage level and appropriate methods to analyze differential expression in glycomics data.

However, differential glycomics expression analysis presents computational and statistical challenges. The complexity of glycans, their variable expression, and high-dimensional glycomics data demand sophisticated analytical tools. Existing methods, while making substantial contributions,^8^^,^^9^ often fall short in addressing all facets of glycomics data, particularly dealing with missing data, normalizing data variance, and accurately determining differential expression with high sensitivity. Further, the current state of the art of *ad hoc* choosing relevant glycan substructures or features for differential expression testing, coupled with the frequent lack of multiple testing correction, renders results across studies incommensurable and likely results in poor statistical decisions, even unconsciously, such as only reporting tests that resulted in significant differences or trying different types of statistical tests and only reporting significant results.

Previous approaches to addressing this gap have highlighted the importance of properly analyzing glycomics data at various levels,^9^^,^^10^ yet have not found widespread adoption and could be further improved by adopting state-of-the-art techniques from differential expression analysis in proteomics and metabolomics, and glycomics-specific advances.

Next to methodological drawbacks in how glycomics data are typically analyzed, the field also lacks domain-adapted workflows for non-standard data types, which have been implemented in transcriptomics, proteomics, and/or metabolomics. These include time-series analyses, multivariate analyses, multi-group analyses, and meta-analyses. We envision a need for glycomics to analyze various data types on the sequence, motif, and motif set levels, which needs to be flexibly incorporated, user friendly, and fueled by state-of-the-art statistical robustness and sensitivity, given the heterogeneous nature of glycomics data and the difficulty of acquiring large datasets.

Previously, we developed a computational ecosystem for analyzing data in the glycosciences with our open-source Python package glycowork.^11^ Next to storing large datasets of species-specific glycan expression and protein-glycan binding, glycowork also provided access to trained deep learning models,^12^^,^^13^ mapping functions between mass, composition, and structure, some motif annotation functionality, biosynthetic network analysis,^14^ and drawing glycans according to the Symbol Nomenclature for Glycans.^15^ As of this writing, glycowork is the most feature-complete solution for glycoinformatics research in Python. Yet, while glycowork exhibited rudimentary approaches to compare samples via heatmaps, we realized that there was no adequate solution for differential glycomics expression analysis within glycowork or any other available package. We also reasoned that the available motif detection and annotation functionality within glycowork could be expanded to approach differential glycomics expression analysis from a multi-faceted angle that allowed for analysis at the sequence and substructure level, to gain readily interpretable insight into glycome dysregulation.

Considering the above-mentioned challenges, we developed a comprehensive computational framework to provide enhanced capabilities for differential glycomics expression analysis and fill this gap. Our platform integrates data normalization methods, sophisticated imputation methods for missing data, (multivariate) differential expression analysis, time-series data analysis, ANOVA, and meta-analysis workflows. All introduced methods can be flexibly used with sequences and motifs, benefiting from the fully automated motif annotation and quantification workflow that we substantially improved here. All methods and workflows are fully integrated into glycowork version 0.8, with user-friendly wrapper functions, facilitating highly optimized workflows that run in seconds on a regular laptop.

We use carefully simulated glycomics data to evaluate the steps of our workflows and real-world glycomics data to demonstrate that insights can be gained rapidly and even from relatively modest sample sizes. We uncover numerous distinct findings from reanalyzing glycomics data, which are statistically robust due to our normalizations and multiple testing corrections. Our objective is to provide more reliable, comparable, and comprehensive tools to facilitate advanced glycomics research, aiding in translating glycomic insights into diagnostic and therapeutic strategies.

## Results

### Automating glycan motif quantification at scale

As glycan motifs often drive glycome functions, for instance by binding proteins,^16^ analyzing glycomics data on a substructure level might offer benefits in interpretation. This is especially true as the same motif might occur in several glycans, presenting a confounder on the sequence level. Thus, motif analysis combines information from biosynthetically related glycans. Related work has shown that this indeed leads to a substantial increase in statistical power, compared with analyzing full sequences.^10^

**Figure 1:**
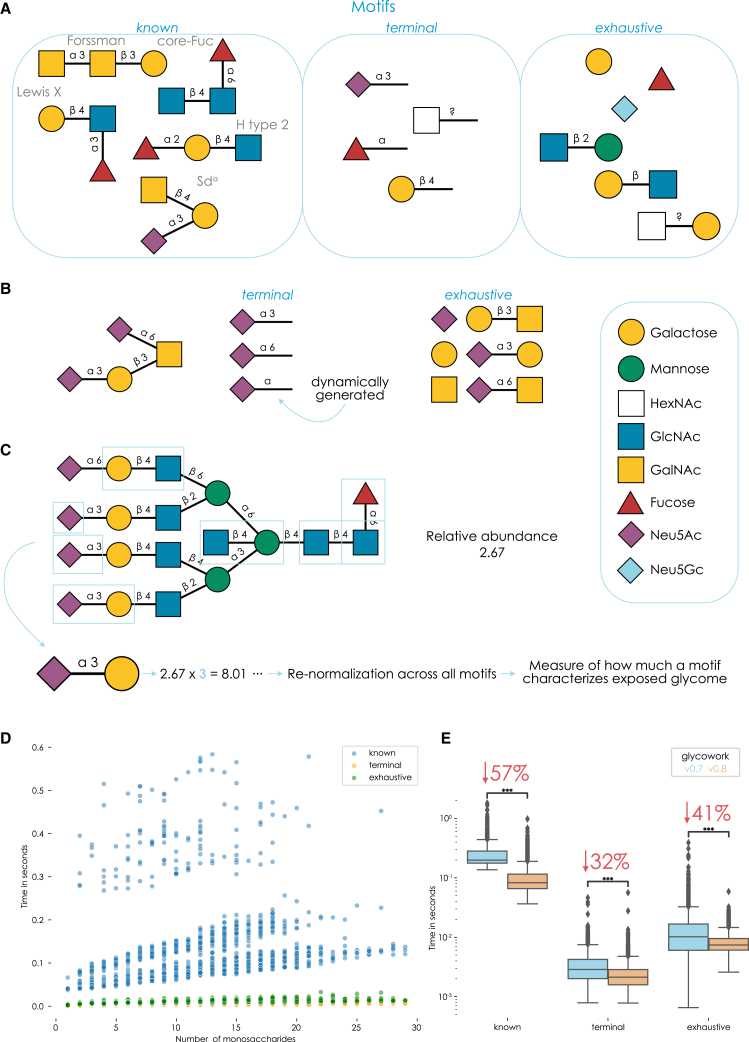
A reworked motif annotation and quantification platform within glycowork (A) Overview of motif types generated within glycowork. (B) Illustration of dynamic motif generation. Annotation functions within glycowork automatically generate more general motifs (e.g., Neu5Acα2-?) and retain them if they capture non-identical information from fully-specified counterparts. (C) Proportional motif quantification. Glycan motifs (blue boxes) are annotated, counted, and used as scaling factors to transform relative abundances into proportions of the cellular surface represented by this motif. (D) Timing motif annotation functions. For all 4,260 human glycans up to 30 monosaccharides stored within glycowork (version 0.8), we timed the *annotate_dataset* portion for each feature_set option (“known,” “terminal,” and “exhaustive”). Differences in the distribution of “known” glycans are dependent on glycan motif density, as more motifs imply more (expensive) subgraph isomorphisms tests. Timing was performed on an Intel Xeon CPU @ 2.20 GHz. (E) Comparing annotation speed between glycowork version 0.7 and 0.8. Similar to (D), annotation functions were timed and compared. Data are depicted as mean values, with box edges indicating quartiles and whiskers indicating the remaining data distribution. Note the logarithmic y axis. Mean differences were tested with two-tailed Welch’s t tests followed by Benjamini-Hochberg correction. The average percent decrease in runtime is shown for each keyword. All glycan structures or motifs in this work are depicted via the Symbol Nomenclature for Glycans (SNFG), drawn using GlycoDraw.^15^ ∗∗∗, p < 0.001; ∗∗, p < 0.01; ∗, p < 0.05; n.s., p > 0.05.

Our glycowork Python package already contained several workflows for automatically annotating glycan motifs.^11^ However, for this work, and included in glycowork version 0.8, we have completely refactored our approach to motif quantification to be more performant, comprehensive, and proportional (Figure 1).

We argue for the value of a standardized vocabulary of assayed glycan motifs, which still retain flexibility for new sequences and various degrees of structural annotation (e.g., uncertain linkages). Motif annotation within glycowork comprises three keywords, which are in general use across glycowork functions and that can be mixed and matched at will (Figure 1A). 154 manually curated motifs (e.g., “LewisX” or “Sda”) can be accessed under the “known” keyword, while the keywords “terminal” and “exhaustive” generate motifs based on provided sequences. The former catalogs all occurring non-reducing end monosaccharides and their linkages, while the latter counts all mono- and disaccharides within a glycan. As discussed in earlier work,^11^ this dynamic calculation of motifs is bounded by observed sequences and balances comprehensiveness with practical considerations (as most theoretical glycan motifs will never be observed). Any motif overlap when keywords are combined is automatically removed within the annotation functions by a herein implemented procedure (STAR Methods). These keywords placed an emphasis on terminal structures and smaller, modular substructures, which are commonly viewed as functional determinants within glycans.^4^^,^^17^

While potent for mediating standardized analyses of differentially expressed substructures that indicated functional consequences, this workflow was occasionally subject to limitations of nomenclature. If sialylation in general increased, this effect would be split between Neu5Acα2-3 and Neu5Acα2-6 when analyzing glycans with the “terminal” keyword. Therefore, we implemented a system (Figure 1B) that dynamically generated the subsuming category (e.g., Neu5Acα2-?) and only retained it if it was not collinear with either specified version (i.e., if it contributed additional information), even if Neu5Acα2-? itself was not present in any glycan. This allowed us to probe more general trends in datasets.

If glycan substructures or features are analyzed in glycomics data, they are usually binarized. This results in, for instance, analyzing “fucosylated” glycans (i.e., glycans with >0 Fuc vs. 0 Fuc). This loses valuable information about motif density. At an equal concentration, an *N*-glycan exposing multiple antennae capped with Neu5Acα2-3 shapes the cellular surface properties more than a glycan with only one antenna containing Neu5Acα2-3. We, therefore, developed a weighting scheme (Figure 1C) that counted each motif (from the above-mentioned keyword classes), retrieved its associated relative abundances, and scaled these abundances by the motif count. Followed by an additional normalization across all motifs, this then provided an indication of the dominance or proportion of a glycan motif across the whole glycome and is a better indicator for biologically relevant changes upon differential expression.

As with any analytical workflow, scalability is important. This will become especially relevant once glycomics is increasingly employed for sample characterization,^18^ for instance, aided by methods to automate data analysis.^13^ We aimed for timescales that would allow for (i) dynamic motif calculations, without need for pre-calculated intermediate results, and (ii) rapid re-running of workflows with different parameters (e.g., different motif keywords), facilitating interactive work. We, therefore, extensively optimized our motif annotation platform to annotate even larger glycans in milliseconds on a regular CPU (Figure 1D). On average, this makes motif annotation in glycowork version 0.8 nearly twice as fast as in version 0.7 (Figure 1E), despite it being more comprehensive and accurate in v0.8. This also translated into gains transcending the differential expression platform we present here and, for instance, eased visualizing motif distributions via heatmaps,^19^ generating biosynthetic networks,^14^ and other motif-level workflows enabled by glycowork.

As motifs have already been chosen in previous versions of glycowork,^11^ we summarize that, here, our advances in general motif analysis comprise (i) faster motif annotation, (ii) more comprehensiveness in the dynamic motif generation (for “exhaustive” and “terminal”), and (iii) proportional motif counting for transforming structure-level data into weighted motif-level data.

### Establishing a robust and sensitive workflow for differential glycomics expression

Regardless of whether the expression of glycan motifs or full sequences is assayed, common issues arise. These include data sparsity, missing data, and heterogeneous data, which inflates the false-negative rate of current differential glycomics expression analyses. This is paired with the current, all-too-common practice of using unadjusted p values to evaluate results, which in turn inflates false-positive rates. We thus set out to conceptualize a workflow that addressed these common challenges, still accommodated motif and sequence analysis, and balanced rigorous statistical procedures with the sensitivity needed for analyzing glycomics data.

**Figure 2:**
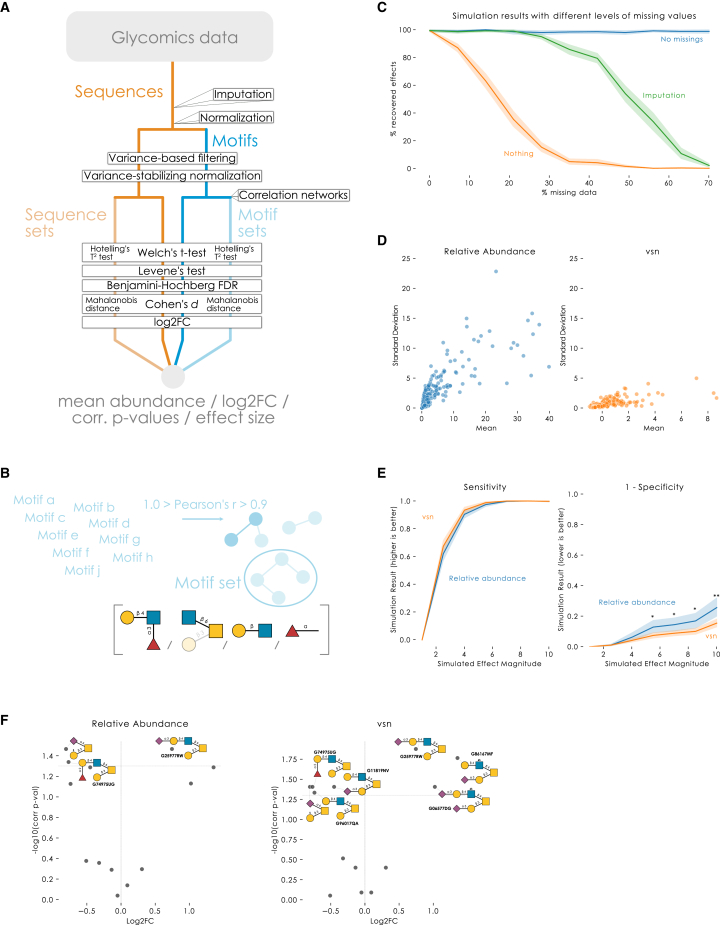
Establishing a statistically robust and sensitive workflow for differential glycomics expression analysis (A) Schematic overview of *get_differential_expression* in glycowork (version 0.8). (B) Overview of motif set construction. If sets = True, *get_differential_expression* will form sets of highly correlated sequences or motifs. These sets are then handled as multivariate tests within *get_differential_expression* and constitute an enrichment analysis. (C) Glycomics data imputation makes the workflow robust to missing data. From 0 to 70% of randomly missing data, we sampled 128 glycans from a Dirichlet distribution (STAR Methods) for 50 experiments per level of missing data, for two groups of ten replicates each (N = 1,000 for each level of missing data). Whether simulated effects were correctly identified was tested with and without data imputation, including a comparison group without missing data. (D) Variance-stabilizing normalization (vsn) minimizes heteroscedasticity. Glycan abundances from nine experimental datasets^20^^,^^21^^,^^22^^,^^23^^,^^24^^,^^25^^,^^26^^,^^27^^,^^28^ were normalized by dividing each abundance by the total abundance. Then, vsn was performed by log-transforming the data and standard scaling to zero mean and unit variance, using *variance_stabilization* in glycowork. (E) vsn improves specificity. Simulations proceeded similar to (C), except without missing data. Instead, the simulated effect magnitude was varied by the concentration parameter of the Dirichlet distribution, scaling from 1 to 10 in seven evenly spaced steps. Statistical significance was established with two-tailed Welch’s t tests and the resulting p values were adjusted using the Benjamini-Hochberg method. ∗∗p < 0.01; ∗p < 0.05. (F) *O*-glycomics data from formalin-fixed paraffin-embedded (FFPE) tissue of healthy and basal cell carcinoma (BCC) samples^20^ were analyzed with *get_differential_expression* to illustrate the impact of vsn-transformation of relative abundances. Results are depicted as volcano plots obtained via *get_volcano* and *annotate_figure*, with manually added GlyTouCan IDs.

This meant that we needed to implement robust methods for (i) data imputation, (ii) data normalization, (iii) statistical testing, and (iv) multiple testing correction, all of which had to be compatible with analyses on the sequence, motif, and motif set levels (i.e., univariate and multivariate statistics). Next to these features, we also implemented effect sizes (Cohen’s *d*/Cohen’s *d*_*z*_ for univariate and Mahalanobis distance for multivariate statistics) and Levene’s test to test homogeneity of variance across groups. Below, we discuss the choice of methods for each element, resulting in a performant workflow for differential glycomics expression analysis (Figures 2A and S2) that we showcase with examples throughout this work.

Next to sequence- and motif-level analyses, we also wanted to support analyzing motif sets. Originally from metabolomics,^29^ set analysis assembles highly correlated metabolites to analyze them as a group, via multivariate statistics, reasoning that a common biological process causes their differential expression. As glycan motifs can have strong biosynthetic relationships (e.g., Sialyl-Lewis × Contains a Lewis × Motif and a Neu5Acα2-3 motif), we hypothesized this to enhance sensitivity. Inspired by metabolomics,^29^ we constructed sequence or motif sets via correlation networks, where two entities with a (positive) correlation between 0.9 and 1 (excluding 1) were connected via an edge (Figure 2B). Each connected component within the network constituted a set that was compared via Hotelling’s T^2^ test (a multivariate extension of the t test^30^), with the Mahalanobis distance to estimate the multivariate effect size.^31^

A recent analysis of imputation strategies for proteomics data identified MissForest,^32^ a Random Forest-based approach, as the current best-in-class method for data imputation.^33^ We thus implemented a version of MissForest within glycowork. This machine learning-based imputation strategy, in contrast to single-value imputations (e.g., replacing all missing values with 0.1) that are commonly used in glycomics data, does not profoundly affect the underlying distribution of glycan abundances and thus should be more robust to artifacts. To test the impact of missing data on the workflow, with and without imputation, we generated simulated glycomics data with 0%–70% randomly missing values (STAR Methods). At 30% missing values, differential expression with data imputation recovered the simulated effects with a sensitivity comparable to analysis without missing values. Employing no imputation decreased the sensitivity to less than 20% (Figure 2C).

Glycomics data are inherently heteroscedastic, displaying increasing variance across measured abundances (Figure 2D). To minimize this phenomenon, and increase statistical power, we performed variance-stabilizing normalization (vsn), log-transforming the data followed by standard scaling to zero mean and unit variance (Figure 2D). Using simulated glycomics data, we compared workflow performance between using relative abundances and the corresponding vsn-transformed values. We observed a significantly lower false-positive rate (1 – specificity) at higher simulated effect magnitudes upon vsn transformation (Figure 2E). We further tested the impact of vsn transformation on workflow performance by reanalyzing *O*-glycomics data from formalin-fixed paraffin-embedded (FFPE) tissue collected from 20 patients with basal cell carcinoma (BCC).^20^ Differential expression analysis using relative abundances as input yielded three significantly changed structures, reflecting a general shift from core 1 to core 2 *O*-glycans as originally reported.^20^ vsn transformation detected four additional significantly changed glycans, showing an increase in sialylated, sulfated core 2 *O*-glycans, and downregulation of their respective biosynthetic precursors (Figure 2E).

For differential expression testing, we chose two-tailed Welch’s t tests (Figure 2A) for their robust empirical performance with small sample sizes and their consideration of potential heteroscedascity in the input data.^34^ In contrast with common misconceptions, t tests do not assume normally distributed input data, but rather normally distributed differences in means (for example), which is obtained even with very small samples.^35^^,^^36^ We still made sure that this assumption holds true in glycomics data and compared it with a nonparametric test (Figure S1), which showed the equivalence of our approach and highlighted the slightly lower rate of false positives that is often achieved with parametric tests.^36^ As further discussed below, an even better approach might lie in generalized linear models (GLMs) and related methods,^37^ yet given typical sample sizes and variances observed today, we believe that our approach is a more fruitful compromise until future technologies will unlock this realm for glycomics data.

**Figure 3:**
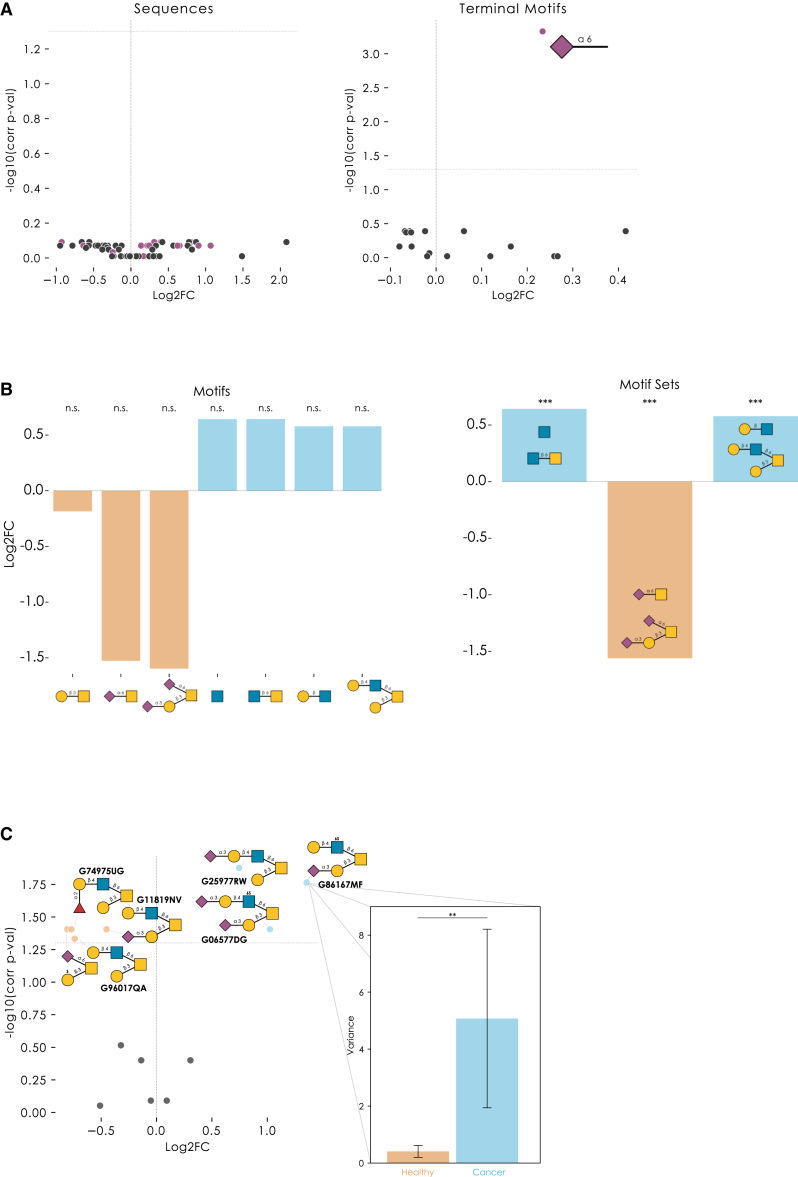
Analyzing differential glycomics expression with a performant workflow (A) Volcano plots comparing workflow sensitivity for sequences and motifs. As a control, random human *O*-glycans from glycowork were assigned to simulated abundances. In the test condition, concentration parameters of structures containing “Neu5Acα2-6” were multiplied by 1.25 (log2 fold-change of ∼0.3). Sequences or motifs containing “Neu5Acα2-6” are labeled purple. N = 5 per condition. (B) The *O*-glycomics data from prostate cancer^21^ were analyzed with *get_differential_expression* at the motif level, with sets = False or = True, to illustrate the enhanced sensitivity by analyzing sets of highly correlated motifs. For sets = False, the seven motifs with the lowest adjusted p values are shown, together with log2-transformed fold changes (cancer vs. healthy). (C) The *O*-glycomics data from BCC^20^ were analyzed with *get_differential_expression* at the sequence level, followed by visualizing significantly differentially expressed glycans via *get_volcano* and *annotate_figure* of glycowork, with manually added GlyTouCan IDs. An inset shows variances of healthy and cancer samples for the only glycan for which Levene’s test for Equality of Variances yielded a significant p_adj_. The bar graph shows the variances, together with a 95% confidence interval estimated by bootstrapping 1,000 times, using sampling with replacement. ∗∗∗p < 0.001; ∗∗p < 0.01; ∗p < 0.05; n.s., p > 0.05. See also Table S3.

To showcase the sensitivity of motif-level analysis, we modified our data simulation to scale the abundance of all glycans containing a motif of interest. Across randomly sampled human *O*-glycans, we scaled the concentration parameter of glycans containing ‘Neu5Acα2-6’ by 1.25 in the test condition, reflecting a modest log2 fold-change of ∼0.3. As expected, no significantly differentially expressed glycans were detected by sequence-level analysis; in contrast, motif-level analysis correctly identified the terminal motif “Neu5Acα2-6” as significantly regulated (Figure 3A). This also made abundantly clear that motif-level effects can be entirely missed when analyzing glycomics data only on the sequence level.

To ensure that our motif annotation and quantification approach was performant, we compared motif abundances derived from GlyCompare^10^ with those we obtained within glycowork on the abovementioned BCC dataset^20^ (Figure S3 and Table S3). While GlyCompare-features did recapitulate the increase in Neu5Acα2-3, glycowork-derived motif abundances yielded a greater number of differentially expressed motifs that reached statistical significance within our workflow. Here, we want to emphasize our end-to-end approach of dynamically quantifying motifs within the analysis, thereby making any pre-processing or post-processing steps unnecessary, which is not the case for most alternative approaches. A loaded table of relative abundances of glycans can thus, literally, be analyzed with a single line of code. This makes our approach inherently more scalable and usable.

As mentioned, our approach of optionally analyzing highly correlated sets of glycan motifs via correlation networks, as in metabolomics,^29^ substantially enhanced sensitivity and robustness to differences in data annotation (such as linkage uncertainties in some structures). As an example, we applied this to 55 prostate cancer *O*-glycomics samples,^21^ in which a motif-level differential expression analysis did not yield any significant differences between tumor and healthy samples after multiple testing correction (lowest p_adj_ = 0.07) (Figure 3B). In contrast, motif set-level analysis revealed biosynthetic clusters of glycan motifs that were significantly dysregulated in cancer, such as a lower level of extended Neu5Acα2-6GalNAc containing glycans and higher levels of extended core 2 *O*-glycans (Figure 3B). This showcased the benefits of motif set-level analysis for, often heterogeneous, glycomics data.

Most statistical tests investigate whether samples differ in their mean values for entities such as glycans. However, it is a well-known fact that diseases, such as cancer, also manifest with a substantial degree of heterogeneity.^38^ Especially bulk approaches, such as glycomics, can be affected by this heterogeneity. Our workflow, therefore, also contained Levene’s test for Equality of Variances that, when significant, indicates that variances are significantly different across the comparison groups. It is important to note that this can occur even in the absence of significant mean differences. Applied to an example dataset of *O*-glycomics data of BCC samples and healthy controls^20^ (Figure 3C), we not only found significant mean differences (sulfated/sialylated glycans upregulated; neutral/fucosylated glycans downregulated) but also one sequence, Neu5Acα2-3Galβ1-3(Galβ1-4GlcNAc6Sβ1-6)GalNAc, which exhibited a highly significant Levene’s test. This supported our choice of Welch’s t test, as the regular t test would not be warranted in such a case of heteroscedasticity. This increased variance of specific sequences in, for instance, heterogeneous diseases such as cancer^38^ could then indicate either increased noise in the regulation of responsible enzymes or cell type subgroups within the bulk sample, which might be identifiable via these glycans. In either case, they are promising starting points for follow-up investigations.

### Accommodating various types of glycomics data and experimental designs

While comparing two groups regarding their glycome is an essential and almost ubiquitous task in analyzing glycomics data, we also wanted to support different data structures. A common problem occurs when more than two experimental groups are compared and the relevant pairwise differences are not known *a priori*. Here, the standard approach would be an ANOVA, followed by *post hoc* tests of glycans/motifs that show any significant differences between groups. We implemented this functionality in the *get_glycanova* function, which triggers Tukey’s HSD (honestly significant difference) test if any ANOVA result was significant. As this test controls the family-wise error rate, the final p values are adjusted for multiple testing. Apart from the statistical testing itself, the *get_glycanova* function benefited from all improvements made to the *get_differential_expression* function, such as our data imputation strategy or variance-stabilizing normalization.

**Figure 4:**
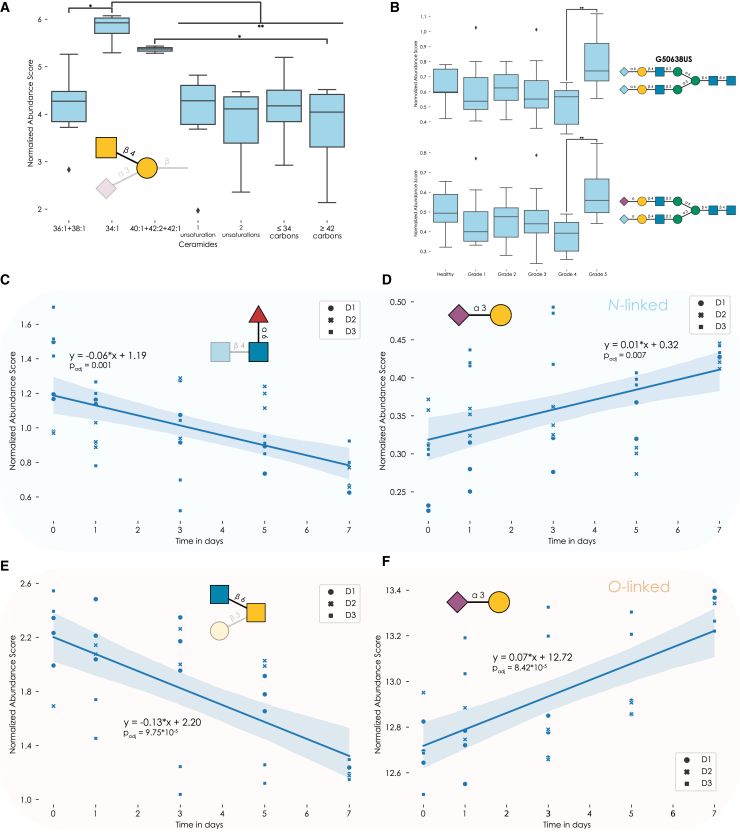
Analyzing multi-group data and glycomics time series data (A and B) We used *get_glycanova* for a motif analysis of a glycosphingolipid dataset^39^ (A) and a sequence analysis of an *N*-glycan dataset^21^ (B). Asking the question whether any glycan motif/sequence was differentially expressed across different ceramide types (A) or cancer grades (B), *get_glycanova* performs an ANOVA and pairwise Tukey’s HSD tests for any significant results. The examples of GalNAcβ1-4Gal (A), part of the Sd^a^ motif, and two Neu5Gc-containing *N*-glycans (B) are depicted as mean values, with box edges indicating quartiles and whiskers indicating the remaining data distribution. (C–F) Representative motifs showing significant changes in *N*-glycans (C) and (D) or *O*-glycans (E) and (F).^40^ We used *get_time_series* from glycowork, with motifs = True, for both *N*- and *O*-glycomes. From the results (Table S4), we chose the core fucose motif (C), terminal α2-3-linked sialylation in *N*-glycans (D), the core 2 *O*-glycan determinant (E), and terminal α2-3-linked sialylation in *O*-glycans (F) as examples. Shown are the respective normalized abundance scores calculated by *quantify_motifs*, with different shapes for points from different donors. Overlayed is a fitted linear regression line, including the 95% confidence interval band, and the regression equation and the Benjamini-Hochberg-adjusted p value of the regression coefficient, derived from a t test. ∗∗∗p < 0.001; ∗∗p < 0.01; ∗p < 0.05; n.s., p > 0.05. See also Table S4.

To illustrate this, we used a previously reported glycosphingolipid dataset^39^ that investigated ganglioside expression from different tissues across various kinds of ceramide portions. Using the *get_glycanova* function, we probed whether any glycan motif was differentially expressed across the different pools of ceramides. This identified elevated motifs, such as the increased expression of GalNAcβ1-4Gal substructures (from the Sd^a^ motif) in short and long, but not middle-sized, ceramide portions (Figure 4A).

Of course, this workflow can similarly be applied on the glycan sequence level, and we used this in a glycomics-adapted ANOVA analysis of an *N*-glycomics dataset of prostate cancer patients^21^ to, for instance, show that several complex *N*-glycans were differentially expressed only between grades 4 and 5 in prostate cancer (Figure 4B), which could indicate their usefulness in patient stratification.

Another example of glycomics data structures would be time-series data, a common setup to test the effect of a drug or monitor a cell differentiation program. While the analysis of such data can be, and often is, reframed as a group comparison (e.g., differential expression of first time point vs. last time point), such an approach decreases sensitivity and may miss transient expression changes.

Instead, in the *get_time_series* function, we chose an approach that fitted an ordinary least squares (OLS) regression to each glycan or motif and performed t tests to evaluate whether the regression coefficients were significantly different from zero. We are aware that this approach may underestimate non-linear trajectories and other effects, but consider it a compromise of currently available data quantities and an improvement compared with current practices. For enhanced functionality and comparability with the methods presented above, the *get_time_series* function used the same data imputation, normalization, motif quantification, and multiple testing correction strategies employed in our other functions.

To illustrate the superior sensitivity and usefulness of this analytic approach, we chose an available glycomics dataset from the literature, in which the differentiation of human monocytes to macrophages was monitored by *N*- and *O*-glycomics time-series data.^40^ Analyzing these two time-series datasets with our workflow resulted in a large number of significantly changing glycan sequences and motifs during this process (Figures 4C–4F and Table S4), despite our more rigorous workflow that included multiple testing correction.

One of the most prominent results for *N*-glycans was the decrease of core fucosylated structures over time (Figure 4C), also noted by the authors of the original study. However, the much lower p value, compared with the t test comparing day 0 and day 7 from Hinneburg et al., clearly showcased the increased sensitivity of our approach. Therefore, while the original authors did report no significant changes in *N*-glycan sialylation, we also noted a significant increase in, specifically α2-3-linked, Neu5Ac among *N*-glycans (Figure 4D).

For *O*-glycans, we first recapitulated the major finding of Hinneburg et al.,^40^ a decrease in core 2-containing structures (Figure 4E). We then went on and further demonstrated a significant increase in α2-3-linked Neu5Ac (Figure 4F), mirroring its increase in *N*-glycans and hinting at a general upregulation of this motif in human macrophages upon differentiation.

We recognize that non-linear expression trajectories might well be possible and relevant for glycomics data and thus also allow for investigating polynomial fits to time series data with the “degree” keyword argument to *get_time_series*. We demonstrated the usefulness of this with data from free milk oligosaccharides^41^ (Figure S4), which often exhibit non-linear trajectories throughout lactation that can be better captured with polynomial functions. Future work could increase the repertoire of *get_time_series* further, to accommodate more complex temporal expression patterns.

### Applying the differential expression workflow to a cancer *O*-glycomics meta-analysis

On the one hand, substantial heterogeneity in glycan expression and different measurement techniques can make cross-study comparisons hard. Yet, on the other hand, increasing data collection capabilities have resulted in a growing set of glycomics datasets targeting the same underlying disease, such as different forms of cancer. Following more mature fields, we hypothesized that combining results from different studies and different patient cohorts could thus allow for more reliable estimates of conserved changes in a disease glycome. The state-of-the-art approach for this procedure is encompassed in the method family of meta-analyses, in which systematic literature searches are followed by procedures to combine effect sizes across studies.

To accommodate this growing potential in the field of glycomics, we have implemented fixed effects and random effects meta-analysis^42^ capabilities into glycowork version 0.8, which were designed to seamlessly work with the differential expression methods mentioned above and can be used to readily combine a series of dataset analyses with the *get_meta_analysis* function.

We then set out to demonstrate the potential of this functionality by performing an explorative mini meta-analysis of pan-cancer *O*-glycomics data from human patient samples (see STAR Methods for details), as there are already at least systematic review-type of analyses for pan-cancer *N*-glycomics data,^43^ yet not for *O*-glycomics data. Surveying the current literature on such *O*-glycomics studies, in which the underlying data have been published as well (essential for re-analysis with our approach), resulted in nine publications with eleven cohorts (total N = 223) from a variety of cancer types.

While there are many invaluable reviews about *O*-glycan dysregulations in cancer,^44^^,^^45^^,^^46^ these mostly comprise manual and subjective collations of various findings across the years. This is certainly valuable, yet lacks the technical rigor of an actual meta-analysis, especially as all the individual findings that led to these collective evaluations came from very heterogeneous data analysis approaches and, due to the lack of multiple testing correction, would not have been findings in many cases in rigorous analyses.

**Figure 5:**
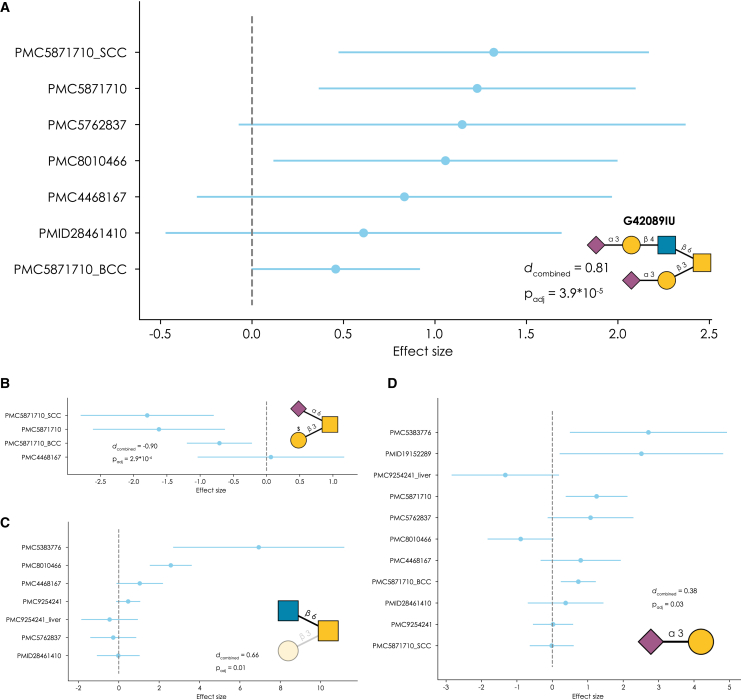
Meta-analysis of cancer *O*-glycomics data using *get_meta_analysis* in glycowork For 11 patient cohorts (9 studies, N = 223) of *O*-glycomics data from tumor and healthy tissue of gastric, skin, liver, prostate, colorectal, and ovarian cancer, we used *get_differential_expression* on the sequence (A) and (B) and motif (C) and (D) levels to obtain effect sizes and their variances. A fixed-effects meta-analysis via *get_meta_analysis* yielded combined effect sizes (Cohen’s *d*) and adjusted p values for each sequence or motif (Table S5). Shown are Forest plots of representative sequences (Neu5Acα2-3Galβ1-4GlcNAcβ1-6(Neu5Acα2-3Galβ1-3)GalNAc, A; GalOSβ1-3(Neu5Acα2-6)GalNAc, B) or motifs (core 2 *O*-glycans, C; Neu5Acα2-3Gal, D), also derived from *get_meta_analysis*. For each study reporting this sequence or motif, the derived effect size and its 95% confidence interval is shown. Further, the combined effect size and the associated adjusted p values are shown. See also Table S5.

Analyzing the effect sizes of differentially expressed glycans and glycan motifs across all these datasets allowed us to reveal which glycan sequences and motifs in fact were consistently dysregulated across the types of cancer included in this analysis (Table S5). The most consistently dysregulated sequence in cancer was Neu5Acα2-3Galβ1-4GlcNAcβ1-6(Neu5Acα2-3Galβ1-3)GalNAc, always being upregulated in tumor samples, albeit with considerable heterogeneity in its expression (Figure 5A). Overexpressed sequences such as this could, in the future, become general purpose cancer biomarkers as well as targets for recruiting anti-tumor proteins.^47^ Importantly, structures such as this one (α2-3-sialylated core 2 *O*-glycans) have been recently identified to define cancer stem cell populations within breast cancer.^48^

Conversely, other sequences such as GalOSβ1-3(Neu5Acα2-6)GalNAc were downregulated in all studies in which they were identified (Figure 5B). It also speaks to a more general trend we have noticed throughout this work, that differentially expressed glycans often were relatively lowly abundant structures, which further hampers this detection due to greater relative variance. Importantly, however, this does not mean that we find sulfated glycans in general to be downregulated in the case of cancer, as other structures, such as Neu5Acα2-3Galβ1-3(Galβ1-4GlcNAc6Sβ1-6)GalNAc, were found to be significantly upregulated (*d*_combined_ = 0.6; p_adj_ = 0.04) and other researchers also identified specific upregulated sulfated *O*-glycans in cancer.^49^

Here again, an analysis of the underlying motifs might shed light on patterns of dysregulation that would not be apparent at the individual sequence level, such as the overexpression of core 2 *O*-glycans in cancer (Figure 5C), which is a known finding.^20^^,^^22^ Another example of this can be found in sialylation. While Neu5Ac in general,^50^ and the sialyl-Tn (Neu5Acα2-6GalNAc) sequence in particular,^51^ are found to be upregulated in cancer, we find this picture to be somewhat more complicated.

While Neu5Acα2-3 is indeed widely upregulated in cancer *O*-glycomes (Figure 5D), total Neu5Acα2-6 is actually downregulated, on average, in reported cancer glycomics data (Table S5). The reason for this is 2-fold: (i) sialyl-Tn is often not reported in cancer *O*-glycomics data (only 6 of our 11 cohorts even report its expression in any sample, including several cohorts with basically identical mean values of sialyl-Tn across groups), and (ii) di-sialyl-T antigen (Neu5Acα2-3Galβ1-3(Neu5Acα2-6)GalNAc) was consistently downregulated in the reported datasets (*d*_combined_ = −0.5; p_adj_ = 0.01) (Table S5). While sialyl-Tn did exhibit a moderate upregulation (*d*_combined_ = 0.3), this did not reach statistical significance (p_adj_ = 0.68) (Table S5). As a consequence, the total pool of Neu5Acα2-6 containing glycans registered as downregulated on average. While we acknowledge that these structures are predominantly biosynthesized by different enzymes^52^ (ST6GALNAC1 for sialyl-Tn and ST6GALNAC3/4 for di-sialyl-T), the fact remains that the number of Neu5Acα2-6 epitopes that are exposed on the cell surface seems to decrease in cancer on average, shifting toward Neu5Acα2-3, at least according to currently published glycomics data. Insights such as these are important reasons for conducting such meta-analyses across the entire measured glycome and at the motif level.

Overall, we conclude that the current prevalence of available *O*-glycomics data from cancer samples is lower than commonly believed. Especially given (i) the high heterogeneity within and between studies, (ii) the fact that many cancer types do not have a single publicly available dataset, and (iii) the potential for biomarkers, given that even here there were some clear signatures, we urge the community to collect, and deposit, more *O*-glycomics data of this type.

## Discussion

Our goal here has been to establish a comprehensive, user-friendly, and potent platform for the comparative analysis of glycomics data at various levels of resolution. Accessible via wrapper functions from glycowork, we aim to provide experimentalists as well as bioinformaticians with easy access to state-of-the-art tools for analyzing glycomics data. Throughout this work, we have demonstrated that the workflows we developed for this are fast, robust, sensitive, and rigorous. We are also enthusiastic to note that our presented workflow yielded numerous results from re-analyzing glycomics datasets for which the original authors either did not detect significant effects or for which most to all reported effects became non-significant after correcting for multiple testing. This showcases the gains that can be made in analyzing glycomics data with a dedicated workflow, despite the relatively modest sample sizes that are commonly collected today.

We note that, while we designed this workflow for analyzing glycomics data with structural resolution (linkages, etc.) obtained from typical MS/MS glycomics data, *get_differential_expression* and all other functions also can be used if glycans have only been assigned at the compositional level (or even just the *m/z* level). Except for the motif functionality, all other benefits presented herein would then still be applicable. The same applies to glycan nomenclatures, as the structure-level workflow is compatible with WURCS, GlycoCT, and any other nomenclature that can be entered into a spreadsheet.

Since glycowork, and our workflow by extension, was designed for glycans written in the International Union of Pure and Applied Chemistry (IUPAC)-condensed nomenclature, the motif-level workflow requires glycans written in IUPAC-condensed formats. We do note that this widespread nomenclature is in active use in most major databases, as well as in the community. Any deviations in formulating IUPAC-condensed glycans can be remedied with glycowork internal functions (*canonicalize_iupac*^15^), while we direct users to the great number of nomenclature converters (glypy,^53^ GlycanFormatConverter,^54^ etc.) for the case that motif-level analysis is desired but only non-IUPAC-condensed formatted glycans are available. Analogously, the IUPAC-condensed notation of motifs of interest (e.g., dysregulated in disease) from our workflow can then be used to interface with commonly used databases in glycobiology, such as GlyCosmos.^55^

While we consider sequence motifs in this work, in principle our workflows are also amenable for analyzing glycans that are featurized in another manner. Examples of this include extracting atomic features of glycans (e.g., charge distribution), which can be retrieved via glyLES,^56^ or graph features of glycans (e.g., betweenness centrality), which can be calculated within glycowork. As glyLES is also supported within glycowork, all these, and other conceivable featurization methods, provide future opportunities to further explore differential expression of glycan features. We also note that other motif libraries exist^57^ (e.g., or https://glycomotif.glyomics.org/glycomotif/GGM) and, as the choice of motifs was already performed during the conception of glycowork^11^ and not the subject of the work here, we encourage researchers to explore these resources in the context of motif-driven analyses. Future work may include the addition of some of the larger motifs into our “known” category, if they are common enough to be relevant, as any motif shorter than a trisaccharide is, by definition, contained within the “terminal” and “exhaustive” categories. We also emphasize the value of the positional encoding our “known” motifs contain, such as the distinction between internal and terminal LacNAc chains, which is not routinely the case for most major motif services available elsewhere.

We are hopeful that future work will add ontology-based enrichment analyses, similar to Gene Ontology (GO) term enrichments, which have, for instance, found use in glycosylation-related transcriptomics.^58^ Given the complex relationships between motifs, this could further boost sensitivity and interpretability. In principle, outputs from *get_differential_expression* could be used to achieve such an analysis as well as other downstream analyses, providing a modular workflow. We note that classical hypergeometric tests, such as for GO term enrichment, are difficult in the case of glycomics, as the number of possible features depends on the data. While, for example, transcriptomics can assume that, in principle, all transcripts could be observed, an *N*-glycomics dataset can neither observe glycolipid nor *O*-glycan motifs (and vice versa). This is especially apparent in our case of “exhaustive” or “terminal” motifs, as the motifs are bounded by the observed sequences and it is not meaningful to calculate the number of theoretically possible motifs, as this would be orders of magnitude larger than the number of realistic motifs. In small parts, our workflow already makes use of subsumptions to tease out higher level hierarchical enrichments, such as our dynamic process of generating motifs with uncertainties that subsume their specific motif counterparts, yet we think this can be substantially strengthened in the future.

While the work presented here focuses on the analysis of glycomics data, we envision that appropriate data transformations could also make data from glycoproteomics amenable to analysis by the herein developed workflow, yielding insights into system-wide changes in glycosylation.

One part of the difficulty in analyzing glycomics data lies in the scarcity of data, usually caused by the time-intensive analysis of mass spectrometry data, particularly from heterogeneous cancer data. We envision that efforts to automate this analysis bottleneck, such as our recently presented CandyCrunch platform,^13^ will lead to an influx of glycomics data, which will not only further enhance the usefulness of the herein presented methods but also facilitate more meta-analyses and conclusions of common dysregulations in the future. In general, the reported meta-data of glycomics data often are sparse at best, which may stymie the determination of eligibility of a study for inclusion into a meta-analysis. We are hopeful that coordinated efforts to implement the MIRAGE (minimum information required for a glycomics experiment guidelines)^59^ system will improve meta-data reporting. Once meta-data will be reported consistently, specialized methods for capitalizing on experiment-specific characteristics (e.g., improving data imputation based on error/variance source) will be possible and might further increase sensitivity and specificity.

As a corollary to this point, we are also optimistic that future improvements of the methods presented in this work could include support for more elaborate experimental designs. Our current main differential expression analysis only allows for two defined groups of comparisons, whereas in principle users could be interested in more sophisticated features such as including interaction terms or mixed-effects models. While this is established in fields such as transcriptomics,^60^^,^^61^ we concluded that the currently existing data acquisition practices in glycomics place this beyond the scope of the current work but remain optimistic that future methodological improvements will facilitate more complex studies.

### Limitations of the study

Our meta-analysis only included a modest number of datasets, and only focused on *O*-glycans, restricting the conclusions that can be drawn about conserved glycan changes in cancer. Additionally, our time-series and polynomial modeling approaches may miss complex non-linear trajectories in glycomics data. While powerful, the computational workflows presented rely on currently available sample sizes and data complexity, constraining some aspects like mixed-effects models. Future advances in data quantity and complexity will allow further method expansion. Overall, this work makes important steps in robust glycomics analysis, but remains limited by available data.

## STAR★Methods

### Key resources table

**Table:** 

REAGENT or RESOURCE	SOURCE	IDENTIFIER
**Deposited data**		

N-glycomics from colorectal cancer	Sethi et al.^62^	N/A
O-glycomics from colorectal and liver cancer	Madunić et al.^22^	N/A
O-glycomics from colorectal cancer	Robbe-Masselot et al. ^23^	N/A
O-glycomics from gastric cancer	Adamczyk et al.^25^	N/A
O-glycomics from gastric cancer	Jin et al. ^27^	N/A
O-glycomics from liver cancer	Hinneburg et al.^28^	N/A
O-glycomics from ovarian cancer	Vitiazeva et al.^63^	N/A
N- and O-glycomics from prostate cancer	Kawahara et al.^21^	MSV000083727
Glycosphingolipid glycomics from human retina	Sibille et al.^39^	N/A
O-glycomics from skin cancer	Möginger et al. ^20^	N/A
Free oligosaccharide glycomics from human milk	Bao et al.^10^	N/A
N- and O-glycomics from macrophage differentiation	Hinneburg et al. ^40^	N/A
N-glycomics from sickle cell disease	Ashwood et al.^64^	GPST000128

**Software and algorithms**		

Glycowork	Thomès et al.^11^	https://doi.org/10.5281/zenodo.10039203; https://github.com/BojarLab/glycowork
Differential Glycomics Expression Analysis	This paper	https://doi.org/10.5281/zenodo.10039203; https://github.com/BojarLab/glycowork/blob/master/glycowork/motif/analysis.py

### Resource availability

#### Lead contact

Further information and requests for resources should be directed to and will be fulfilled by the lead contact, Daniel Bojar (daniel.bojar@gu.se).

#### Materials availability

This study did not generate new unique reagents.

#### Data and code availability

• Data curated or generated here can be found in the supplementary tables as well as stored as internal datasets within glycowork. Publicly available datasets used are listed in the key resources table.
• All relevant code is integrated into glycowork (version 0.8).^65^ An archival DOI is provided in the key resources table.
• Any additional information required to reanalyze the data reported in this paper is available from the lead contact upon request.

### Method details

#### Data simulation

Working under the assumption of normalized glycomics data, in which all glycans of a sample sum up to a relative abundance of 100 and only positive abundances are permitted, we modeled this type of data via a Dirichlet distribution, as a specific case of a multivariate distribution in which the components (features) are positive and sum to a constant (100 in this case). Our default simulation set-up included 118 glycans, for which we used concentration parameters according to the experimental relative abundances of the first ‘healthy’ replicate from the *N*-glycan dataset found in Ashwood et al.^64^ (Table S4 in Ashwood et al.; Table S2 here). Ground truth effects were added by duplicating a subset (default n = 10 per group of effects, i.e., up- or downregulation) of abundances at random, while scaling the concentration parameter of the test condition by the desired effect magnitude. In each run, random glycan structures were sampled from SugarBase^11^ within glycowork and assigned to the simulated abundances. Random sialylated structures were assigned to the group of upregulated glycans, while random fucosylated structures were assigned to the group of downregulated glycans.

For evaluating the motif-level workflow, the abundance of each glycan containing the motif of interest was scaled by 1.25.

For evaluating imputation strategies, we varied the proportion of randomly missing values from 0 to 70% in ten linearly spaced steps, using a concentration parameter scaling factor of 5.

For evaluating normalization strategies, we varied the concentration parameter scaling, a measure of simulated effect strength, from 1 to 10 in seven linearly spaced steps.

The stability of the simulation results was evaluated by performing the imputation strategy test using relative abundances from different experimental datasets as the concentration parameters for the Dirichlet distribution (Figure S5)

#### Dataset collection

All glycomics datasets were gathered from the academic literature and were present as supplementary tables in the original articles. We uniformly formatted all these tables for our analyses and they can be found in Table S1. Glycan sequences were reformatted into IUPAC-condensed via the *canonicalize_iupac* function from glycowork,^11^ followed by a manual inspection. Only glycans where at least a defined core topology (including floating substituents) was annotated were considered for our analyses. We reiterate that this is not necessary for sequence-level analysis with our presented methods but, as we also wanted to explore motif- and motif set-level analyses, we focused on uniform data standards.

For the meta-analysis, we used PubMed, Google Scholar, and Google to search for relevant articles with keyword combinations such as “cancer+glycome” or “tumor+glycome”. All articles were screened and only articles with (i) *O*-glycomics data, (ii) data from tumors, (iii) data from healthy controls, (iv) at least two samples per condition, and (v) at least defined glycan topologies were retained. Then, these articles were processed as described above. The resulting data can be found in Table S1.

#### Data normalization

As a first step, we removed variables which had missing values in more than half the replicates, with the rest of the missing values being subject to data imputation described below. We note that this initial removal step can be customized with a keyword argument (‘min_samples’, specifying how many replicates per group need to exhibit a nonzero value for retaining the glycan). The standard normalization procedure employed here was division of each abundance by the total abundance of a sample, essentially normalizing to a total of 100 per sample. Variance stabilizing normalization, used prior to statistical comparisons, included log-transformation of the data and standard scaling to zero mean and unit variance.

#### Data imputation

Data imputation proceeded via the MissForest^32^ approach: all missing values were recorded and then first replaced by the sample median. Then, for each sample, a Random Forest Regressor (default parameters as defined in scikit-learn, version 1.2.2) was trained on all other samples to predict the values at the positions holding missing values. This imputed dataframe was then used in another round of model training and prediction, for a total of five iterations. Finally, we added a small constant (1e-6) to ensure that no zero values were present in the final dataset.

#### Motif extraction and quantification

Motifs were extracted and quantified at three different levels, informed by domain knowledge and accessible via keywords to the *get_differential_expression* function within glycowork, which can be freely combined.

*known*: A set of 154 named motifs (e.g., “LewisX”), defined within glycowork and Table S6, is used to count the number of occurrences within each glycan via subgraph isomorphism tests. Defined monosaccharide positions (terminal/internal/flexible) ensure matching of structures at appropriate positions (e.g., “Oglycan_core1” (i) matches only at the reducing end and (ii) does not double-count core 2 structures as core 1).

*terminal*: For all non-reducing termini, the monosaccharide and its linkage are extracted and all unique combinations are counted in each glycan. Then, in an effort of feature engineering, linkage uncertainties are tentatively introduced (e.g., “Neu5Ac(a2-?)” if “Neu5Ac(a2-3)” is present) to probe whether they result in new/additional information compared to their defined versions. If not (i.e., if they result in identical results), they are removed again. The purpose here is to capture broader enrichments (e.g., enriched in the umbrella term “Neu5Ac(a2-?)”, versus weaker enrichments in the more specified “Neu5Ac(a2-3)” or “Neu5Ac(a2-6)”).

*exhaustive*: All occurring mono- and disaccharide motifs in the provided glycans are counted for each glycan. Similar to the procedure described above for ‘terminal’, we tentatively introduced uncertainties (e.g., “Gal(b1-?)GlcNAc”) to capture broader effects and trends, yet only retained them if they provided additional information.

We then used these counts to arrive at motif relative abundances by forming a weighted sum for each motif. This was achieved by multiplying the relative abundance of each glycan by its motif count, effectively arriving at the representation of how dominantly exposed a given motif is in a sample.

Next, we automatically deduplicated this quantification via the *clean_up_heatmap* function, as sometimes nominally different motifs (e.g., “Neu5Ac”, “Neu5Ac(a2-3)”, “Neu5Ac(*a*2-3)Gal”) can have the same relative abundance distribution (e.g., if the only occurrence of “Neu5Ac” in a dataset is within “Neu5Ac(*a*2-3)Gal”). This was done to reduce the number of statistical tests and hence increase the sensitivity of our analyses. If multiple motifs resulted in the exact same distribution, only one was retained, with the prioritization of named motif > disaccharide > terminal > monosaccharide, guided by the principle of identifying the largest substructure that exhibits this enrichment, which should ease interpretability.

#### Deriving motif abundances from GlyCompare

GlyCompare was implemented via GlyCompareCT.^66^ As the recommended conda installation could not be created in our hands, an alternative approach was taken by manually installing certain packages from the environment list in a new conda environment. First, the GlyCompare version was specified as git+https://github.com/LewisLabUCSD/GlyCompare.git@15b415b. Then, other packages were installed at their required specific versions: pandas = = 1.3.5, bootstrapped = = 0.0.2, lxml = = 4.7.1, scipy = = 1.7.3, and networkx = = 2.4. The basal cell carcinoma data^20^ then was converted into GlycoCT using the structure translation API from GlyConnect.^67^ Once the structural annotation file was prepared along with the original abundance file, as per the GlycompareCT ‘Prepare data’ Step 1a and 1b, GlycompareCT.py was run using the default parameters. The motif_annotation.csv file, mapping the motif codes to WURCS, was then used to convert the motifs into IUPAC-condensed with the GlyCosmos GlycanFormatConverter.^54^ The motif abundance table with converted nomenclature was then analyzed using *get_differential_expression* with motifs = False.

#### Enrichment analysis

To form automated sets that were tested for differential expression via multivariate comparisons, we calculated a pairwise correlation matrix from either sequences or motifs. Then, an adjacency matrix was built by forming a link between two sequences/motifs, if and only if they positively correlated above a set threshold (Pearson’s correlation coefficient; default in this work: 0.9) but below 1. Connected components within the graph described by this adjacency matrix were then used as glycan/motif sets for multivariate comparisons.

#### Differential expression analysis

Using variance-based filtering, all sequences/motifs below a minimum variance cut-off value (default in this work: 0.01, or 1%) were removed before differential expression analysis. Then, the data were split into the sample groups defined for comparison. Log-fold changes were calculated as the log_2_-transformed ratio of group means. In the multivariate scenario, log-fold changes of all set-members were averaged. Then, variance stabilization normalization, with the group-specific means and standard deviations, was used before statistical testing. For the univariate case, two-tailed Welch’s t-tests were used to compare normalized abundances, while Hotelling’s T^2^ test was used in the multivariate case.^30^ Paired samples are also supported within *get_differential_expression*, using paired t-tests and Cohen’s *d*_*z*_ in the univariate setting.^68^ For each comparison, we then further calculated an effect size, Cohen’s *d* for univariate comparisons and the Mahalanobis distance for multivariate comparisons.^31^ Further, in all settings, we also used Levene’s test for equality of variances. In any scenario, all resulting p values were corrected for multiple testing by the Benjamini-Hochberg procedure.

#### ANOVA

Data processing for ANOVA analyses, via the *get_glycanova* function, mirrored those of differential expression analysis above. For each feature, glycan or motif, a linear model was fitted on the abundance via ordinary least-squares regression. This was followed by generating an ANOVA table and the corresponding F statistics. Resulting p values were corrected for multiple testing by the Benjamini-Hochberg procedure. If any ANOVA result reached the significance threshold of 0.05, a Tukey’s HSD (honestly significant difference) test was triggered for all pairwise comparisons of this glycan or motif. Adjusted p values of lower than 0.05 were then retained as significant pairwise differences.

#### Time-series analysis

Data with multiple timepoints were analyzed with the *get_time_series* function, which again exhibited the same data imputation and motif quantification capabilities as the *get_differential_expression* function. Then, for each glycan or motif, an ordinary least squares model was fitted, aiming to explain glycan abundance as a function of time, with a constant intercept. This was followed by t-tests ascertaining whether the slope of the resulting fit was significantly different from zero. If degree >1 was chosen, a polynomial function of the chosen degree was instead fitted to the data. Then, a one-way F-test was performed to test whether the fitted model significantly reduced the residuals compared to an intercept-only model. Resulting p values were corrected for multiple testing by the Benjamini-Hochberg procedure.

#### Meta-analysis via fixed-effects and random-effects models

To calculate effect sizes across different studies, we used the *get_meta_analysis* function within glycowork to estimate combined effect sizes via a fixed-effects model. Here, the individual effect sizes were averaged, weighted by the inverse of their variance. We then also calculated a two-tailed p value for this combined effect size. Within glycowork, effect size variances were calculated within *cohen_d* for Cohen’s *d* and estimated via bootstrapping (N = 1000) for the Mahalanobis distance.

For random-effects models, between-study variance was estimated as τ^2^ by the DerSimonian and Laird method^69^ in *get_meta_analysis*. If a filepath was specified, effect sizes for a given sequence of motifs were plotted in Forest plots across studies with their 95% confidence interval.

For the meta-analysis presented in this work, we used the *get_differential_expression* function to analyze all cancer datasets individually on the motif level (feature_set = [‘known’, ’exhaustive’, ’terminal’]). If the data came from paired samples (e.g., tumor tissue and adjacent healthy tissue from the same individual), we set paired = True when analyzing the data. We then collected the effect sizes and effect size variances for each motif and, for each motif, called the *get_meta_analysis* function with model = ’fixed’ to calculate a combined effect size and resulting p value. Then, all p values were corrected for multiple testing via the Benjamini-Hochberg procedure.

### Quantification and statistical analysis

For statistical analysis, this study used two-tailed Welch’s t-test for univariate and Hotelling’s T^2^ test for multivariate comparisons. Differences in variance were tested by Levene’s test. Pairwise post-hoc comparisons were done with Tukey’s HSD (honestly significant difference) test. All multiple testing corrections were done via the Benjamini-Hochberg procedure. Effect sizes were estimated via Cohen’s *d*/*d*_*z*_ for univariate and the Mahalanobis distance for multivariate comparisons. All statistical testing has been done in Python 3.11.3 using the glycowork package (version 0.8), the statsmodels package (version 0.14) and the scipy package (version 1.11). Data normalization and motif quantification was done with glycowork (version 0.8).

## Supplemental information

Document S1. Figures S1–S 5

Table S1. All curated glycomics datasets used for differential expression analysis, related to STAR Methods

Table S2. Experimental glycan abundances used to establish glycomics simulations, related to Figure STAR Methods

Table S3. Differential expression results using motif abundances gained from GlyCompare or glycowork, related to Figure 3

Table S4. Normalized motif abundances and results from the time series analysis of N- and O-glycomics data of macrophage differentiation, related to Figure 4

Table S5. Combined effect sizes and p-values for O-glycan sequences and motifs from the cancer glycomics meta-analysis, related to Figure 5

Table S6. Description of literature-known motifs used within glycowork, related to STAR Methods

Document S2. Article plus supplemental information
